# Clinical Characteristics of Individuals with Interstitial Lung Diseases and Indication of End-of-Life Care

**DOI:** 10.3390/jcm12237314

**Published:** 2023-11-25

**Authors:** Gabriela Krinski, Larissa Dragonetti Bertin, Heloise Angélico Pimpão, Humberto Silva, Brunna Luiza Tavares, Leonardo Lunardelli, Geovana Alves do Prado, Fabio Pitta, Carlos Augusto Camillo

**Affiliations:** 1 Laboratory of Research in Respiratory Physiotherapy, Department of Physiotherapy, State University of Londrina, Londrina 86038-350, Brazil; gabriela.krinski@hotmail.com (G.K.); larissadbertin@gmail.com (L.D.B.); heloapimpao@hotmail.com (H.A.P.); humberto.s.fit@gmail.com (H.S.); brunnaluizast@gmail.com (B.L.T.); lunardelli.ft@gmail.com (L.L.); fabiopitta@uel.br (F.P.); 2 Department of Rehabilitation Sciences, Pitágoras Unopar Anhanguera University, Campus Piza, Londrina 86041-140, Brazil; 3 Department of Physiotherapy, School of Technology and Sciences, Campus Presidente Prudente, São Paulo State University (UNESP), Presidente Prudente 19060-900, Brazil

**Keywords:** interstitial lung diseases, palliative care, end-of-life care

## Abstract

End-of-life care (EOLC) is palliative support provided in the last 6 months to 1 year of a patient’s life. Although there are established criteria for its indication, few studies describe the clinical and functional characteristics of individuals with interstitial lung diseases (ILD) in EOLC. ILD individuals underwent various assessments, including lung function, exercise capacity (6 min walk test), physical activity in daily life (PADL), peripheral muscle strength, maximal respiratory pressures, body composition, quality of life (SGRQ-I), symptoms of anxiety and depression, dyspnea (MRC scale), and sleep quality. Fifty-eight individuals were included and divided into two groups according to the indication for commencing EOLC (ILD with an indication of EOLC (ILD-EOLC) or ILD without an indication of EOLC (ILD-nEOLC). There were differences between the groups, respectively, for steps/day (2328 [1134–3130] vs. 5188 [3863–6514] n/day, *p* = 0.001), time spent/day carrying out moderate-to-vigorous physical activities (1 [0.4–1] vs. 10 [3–19] min/day, *p* = 0.0003), time spent/day in standing (3.8 [3.2–4.5] vs. 4.8 [4.1–6.7] h/day, *p* = 0.005), and lying positions (5.7 [5.3–6.9] vs. 4.2 [3.6–5.1] h/day, *p* = 0.0004), the sit-to-stand test (20 ± 4 vs. 26 ± 7 reps, *p* = 0.01), 4 m gait speed (0.92 ± 0.21 vs. 1.05 ± 0.15 m/s, *p* = 0.02), quadriceps muscle strength (237 [211–303] vs. 319 [261–446] N, *p* = 0.005), SGRQ-I (71 ± 15 vs. 50 ± 20 pts, *p* = 0.0009), and MRC (4 [3–5] vs. 2 [2–3] pts, *p* = 0.001). ILD individuals with criteria for commencing EOLC exhibit reduced PADL, functional performance, peripheral muscle strength, quality of life, and increased dyspnea.

## 1. Introduction

Interstitial lung diseases (ILD) encompass a heterogeneous group of conditions with similar clinical, radiological, and functional features [1,2,3]. In addition to respiratory impairment, individuals with ILD commonly present systemic manifestations such as reduced participation in activities of daily living, poor exercise tolerance and exercise desaturation, muscle weakness, cough, fatigue, dyspnea, and symptoms of anxiety and depression [4,5]. Following diagnosis, the average life span of patients with the most prevalent ILD, idiopathic pulmonary fibrosis (IPF), varies from 3 to 5 years in different studies, but its clinical course is variable [6,7,8].

The unpredictable course of ILD ma”es its prognosis challenging; therefore, the current guidelines recommend early palliative care for these individuals [7,9]. Palliative care is an approach aimed at improving the quality of life for patients and families facing life-threatening illness-related issues [10]. These interventions are applicable from the early stages of the disease through early identification, assessment, and treatment of symptoms in physical, psychosocial, and spiritual contexts [10]. Palliative care is underutilized in ILD individuals, with referral rates ranging from 0 to 38%, often only occurring in the last month of life [7,9].

Specific end-of-life palliative support is known as end-of-life care (EOLC) and is estimated to occur in the last 6 months to 1 year of a patient’s life [8,11]. The primary goal of EOLC is to promote comfort and improve quality of life until the moment of death [4,6,7]. Patients receiving EOLC require fewer visits to the emergency department and hospitalizations towards the end of their lives, shorter hospital stays, fewer admissions to intensive care units, and in-hospital deaths [6]. There are criteria in the literature for initiating EOLC in ILD individuals, considering factors such as hospitalizations in the past year, resting arterial oxygen saturation (SaO2), exercise capacity, diagnosis of pulmonary hypertension, lung function, and the emergence of new severe comorbidities [8]. However, there is limited current evidence comprehensively describing different clinical and functional characteristics in ILD individuals receiving EOLC.

Understanding the various clinical and functional aspects affected in ILD individuals receiving EOLC would assist in symptom management and improve functionality and quality of life. This study aims to characterize various clinical and functional aspects of ILD individuals with clinical indications to receive EOLC by comparing them with individuals without indications for EOLC.

## 2. Materials and Methods

### 2.1. Study Design and Ethical Aspects

This is a cross-sectional study conducted in the physiotherapy outpatient clinic of the University Hospital of the State University of Londrina (Londrina, Brazil). This study is an analysis of data collected for an ongoing cohort (BELIEVE-ILD #NCT03400839) approved by the local ethics committee of the institution (#2.484.871). Before participating in the study, all participants signed an informed consent form.

### 2.2. Sample Characteristics

Individuals between 40 and 75 years of age with a diagnosis of interstitial lung disease according to international guidelines [3] were included. Individuals with a stable clinical condition (no exacerbations) for at least 1 month before inclusion and without any clinical conditions that could interfere with the assessments (e.g., musculoskeletal limitations, severe or unstable cardiovascular disease, and neuromuscular disease) were included. Individuals whose interstitial lung disease could not be confirmed by pulmonary function testing or who showed characteristics of another lung disease in pulmonary function testing, such as chronic obstructive pulmonary disease, were excluded.

After inclusion, participants underwent the following assessments: medical history, lung function, disease severity, exercise capacity, physical activity in daily life (PADL), functional performance, peripheral and respiratory muscle strength, body composition, health-related quality of life, symptoms of anxiety and depression, and sleep quality. The assessments are detailed below.

### 2.3. Assessments

During the initial assessment, personal and health-related anthropometric data and general health information such as diagnosis duration, home oxygen therapy use, number of hospitalizations in the last year, comorbidities, and medication use were collected.

A complete lung function assessment was performed using spirometry, carbon monoxide diffusion capacity (D_L_CO), and measurements of lung volumes using a whole-body plethysmograph (Vmax, CareFusion, Yorba Linda, CA, USA) [12,13]. Disease severity staging (i.e., I, II, or III) was performed using the Gender–Age–Physiology (GAP) index [14]. The six-minute walk test (6MWT) was conducted to assess exercise capacity according to international standards [14,15]. Objective monitoring of physical activity in daily life (PADL) was performed using an activity monitor (Actigraph wGT3X-BT^®^, ActiGraph LLC, Pensacola, FL USA) [16]. Subjects were instructed to wear the activity monitor on their waist for six consecutive days, for 24 h, including sleeping time. The PADL assessment was considered valid if the subject wore the monitor for at least 8 h/day for at least 4 days [16]. The PADL measurement data were analyzed by the ActiLife^®^ software v6.13.4 (Actigraph, Pensacola, FL, USA).

Functional performance was assessed using the timed up-and-go test (usual protocol), 1 min sit-to-stand test, and 4 m walking speed test [17,18,19]. Reference values, according to Furlanetto et al., were used [20]. The peripheral muscle strength of the pectorallis major, latissimus dorsi, triceps, and biceps brachii muscles, as well as the quadriceps femoris, was evaluated using maximum voluntary isometric contraction (MVIC) with a dynamometer (EMG System^®^, Sao Paulo, Brazil) [21]. The positions adopted for each test were determined according to the muscle function tests [22].

Respiratory muscle strength was assessed using maximal inspiratory and expiratory pressures (PImax and PEmax, respectively) with a digital manovacuometer (MVD 300, Globalmed, Porto Alegre, Brazil) [23,24]. Body composition was assessed using a bioelectrical impedance analysis (Model 310, Biodynamics, Shoreline, WA, USA), and data on the percentage of body fat, percentage of lean mass, metabolic rate, and body mass index (BMI) were collected [25].

Quality of life was assessed using the Saint George’s Respiratory Questionnaire specific for pulmonary fibrosis (SGRQ-I), validated for ILD individuals [26,27]. The dyspnea sensation related to physical activity was evaluated using the modified Medical Research Council scale (MRC) and the University of California San Diego Shortness of Breath Questionnaire (UCSD-SOBQ) [28,29,30]. Symptoms of anxiety and depression were assessed using the Hospital Anxiety and Depression Scale (HADS) [31,32]. The Pittsburgh Sleep Quality Index (PSQI) was applied to assess sleep quality, and the Epworth Sleepiness Scale (ESS) was used to assess daytime sleepiness [33,34].

### 2.4. Indication for End-of-Life Care

Participants were allocated to two groups according to the indication to commence EOLC following internationally accepted criteria (Table 1) [8]. Individuals with an indication of EOLC (i.e., the ILD-EOLC group) had at least two of the criteria described in Table 1. The results of the clinical testing were compared with the data of individuals without an indication to commence EOLC (the ILD-nEOLC group).

### 2.5. Statistical Analysis

The statistical analysis was performed using SAS^®^ OnDemand for Academics software (https://welcome.oda.sas.com, accessed on 1 June 23, SAS, Cary, NC, USA). Data normality was assessed using the Shapiro–Wilk test. Variables were described as the mean and standard deviation or the median and interquartile range. Categorical data were reported as frequencies. To compare data between the ILD-EOLC and ILD-nEOLC groups, unpaired t-tests, Mann–Whitney tests, Fisher’s exact tests, and chi-square tests were conducted, depending on the data distribution. To confirm if the allocation within the ILD-EOLC group was associated with an increased risk of 1-year mortality, an exploratory analysis using the Kaplan–Meier method with the log-rank test was conducted. Furthermore, to ascertain the impact of the outcomes on mortality, both univariate and multivariate logistic regression analyses were carried out. Statistical significance was determined at *p* < 0.05.

## 3. Results

**Figure 1:**
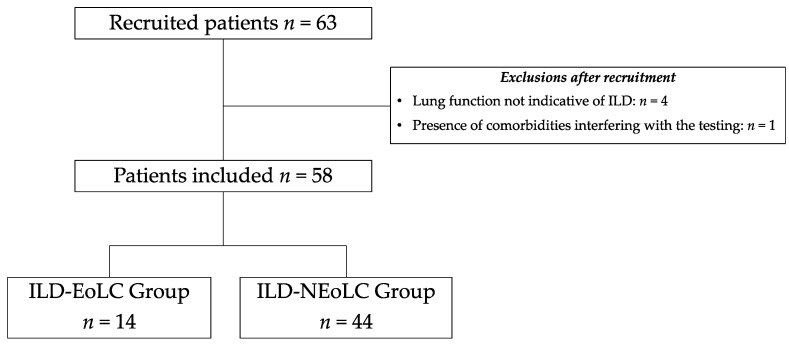
Recruitment and inclusion flowchart of study participants. ILD = interstitial lung disease; EOLC = end-of-life care; nEOLC = no indication for end-of-life care.

Sixty-three individuals were initially recruited for study participation. Of these, 5 were excluded, resulting in a total of 58 individuals (the ILD-EOLC group *n* = 14 and the ILD-nEOLC group *n* = 44) in the data analysis (Figure 1).

Table 2 describes the characteristics of the individuals in both groups. No differences were found regarding sex, age, the time of diagnosis, drug treatment, and the presence of comorbidities. The most common comorbidities in the ILD-EOLC group were gastroesophageal reflux, coronary artery disease, and type II diabetes mellitus. Among the individuals in the ILD-nEOLC group, the most common comorbidities were gastroesophageal reflux, depression, and type II diabetes mellitus. From the included sample, only two patients were following regular exercise training programs before inclusion. After the commencement of the study, 16% of patients in the ILD-nEOLC group and 50% in the ILD-EOLC group were enrolled in regular exercise training programs.

The most prevalent diagnosis in the ILD-EOLC group was idiopathic pulmonary fibrosis (IPF), while most individuals in the ILD-nEOLC group had a diagnosis of ILD associated with connective tissue diseases. The individuals in the ILD-EOLC group had a worse disease stage compared to the ILD-nEOLC group and a higher frequency of home oxygen therapy use. Furthermore, more deaths occurred within 1 year after inclusion in the study in the ILD-EOLC group. Importantly, none of the individuals included in the study were receiving or had ever received palliative care or end-of-life care, even in the group with an indication for that (ILD-EOLC).

Table 3 demonstrates a comparison of the outcomes used in the classification for the indication of EOLC. As expected, the individuals in the ILD-EOLC group exhibited worse results in nearly all outcomes, except for the presence of pulmonary hypertension.

The comparison of the clinical outcomes investigated in this study can be found in Table 4. Except for body composition, respiratory muscle strength, symptoms of anxiety and depression, sleep quality, and daytime sleepiness, the individuals in the ILD-EOLC group exhibited worse results in all the other outcomes. The Kaplan–Meier analysis with the log-rank test revealed a significant difference in mortality between the groups (log-rank: 0.004). The univariate logistic regression analysis identified D_L_CO (<0.001) and the number of steps per day (0.01) as factors associated with mortality. However, in the multivariate analysis, none of the outcomes exhibited a statistically significant difference.

## 4. Discussion

The findings of this study show that individuals with ILD with an indication to commence EOLC present worse levels of daily physical activity, functional performance, peripheral muscle strength, quality of life, and dyspnea. There were no significant differences in respiratory muscle strength, body composition, sleep quality, daytime sleepiness, and symptoms of anxiety and depression.

It is expected that individuals in EOLC have a more severe disease, a poorer overall health status, and a worse prognosis [6,9,35,36]. Indeed, in the current study, the majority of individuals in EOLC had a GAP staging ≥ 2 (i.e., 93%), had 4 to 6 comorbidities (50%), and were using corticosteroids and immunosuppressants (35%). Furthermore, 78% of individuals in the ILD-EOLC group died within one year of follow-up, confirming the severity of the disease in this group. However, some aspects of this severity need to be analyzed in further detail.

The main criteria that led to the allocation of our individuals into the ILD-EOLC group were hospitalizations, reduced lung function, and peripheral oxygen saturation < 88% at rest. Previous studies have shown that a reduction in the 6 min walk test (6MWT) distance is directly associated with mortality [37,38,39]. Surprisingly, although individuals in this group had a shorter 6MWT distance, only one patient in the ILD-EOLC group met the criterion for reduced exercise capacity (i.e., <212 m) [8]. The 212 m cut-off may be too low for the Brazilian population, as it has been shown that Brazilian individuals with chronic respiratory diseases cover longer distances in the test [40].

Since we observed heterogeneity in the indications for EOLC in these individuals, it is possible to question whether these factors can be used to assess a patient’s overall condition. To the best of our knowledge, the present study is the first to evaluate a wide range of physical and functional factors in individuals with ILD with an indication to commence EOLC. In fact, it was possible to observe that several characteristics showed some degree of impairment in these individuals. For example, the individuals in the ILD-EOLC group had lower levels of physical activity in all the investigated domains, except for time spent sitting. Although patients in the ILD-nEOLC group already present a reduction in the number of daily steps, the further reduction in the ILD-ELOC group is worrisome and highlights how little these patients are moving during their daily lives. Previous evidence pointed to an increased risk of mortality with reduced levels of physical activity in ILD [41]. Also, Wallaert et al. suggested that patients with less than 3287 steps/day are at a higher risk of mortality [42], corroborating the findings in our study. The reductions found in peripheral muscle strength and functional performance have previously been associated with mortality in other respiratory diseases [43,44]. In the present cohort, the patients in the ILD-EOLC group presented a lower 1 min sit-to-stand outcome (35% predicted). Although the test measures functional performance, it can be used as a surrogate for muscle endurance and implies that muscle function, not only strength, is compromised in this group of patients. Given the fact that these outcomes were also worse in the ILD-EOLC group, it is possible to suggest that future studies with the power to analyze such associations investigate their connection with mortality in individuals with ILD.

Other domains related to a patient’s perception of their health status showed impairments in the ILD-EOLC group. There was a statistically significant reduction in quality of life, mainly in the domains of activity and disease impact. This result is not surprising, as there is evidence of a worsening quality of life in ILD-EOLC individuals [4,6,35,36]. Furthermore, in the present study, individuals in the ILD-EOLC group had worse scores on both tools used to assess dyspnea (i.e., MRC and UCSD-SOBQ). Dyspnea affects up to 94% of respiratory individuals in EOL care and is usually more common and intense in ILD than in oncology individuals [45].

A controversial finding in our study was the absence of worsened symptoms of anxiety and depression in the ILD-EOLC group. There is evidence that the presence of anxiety and depression in ILD individuals may be intensified at the end of life due to a high symptom burden, worsening functionality, and difficulty in performing activities of daily living [4,6,7,8,35,36]. A likely explanation for this difference is that 42% of the EOLC individuals were taking medications to control these symptoms, while in the ILD-nEOLC group, only 27% were. Similarly, we did not find a significant difference between the groups for sleep quality and daytime sleepiness, as both groups had poor sleep quality (PSQI ranging from 5 to 10) and no daytime sleepiness (ESS ≤ 10) [33,34]. These results suggest that the reduction in sleep quality does not depend on disease severity and that sleepiness does not impact ILD individuals to a large extent [46,47].

The results of the present study should be interpreted with caution due to several limitations, including the small sample size, particularly in the EOLC group, and the use of internationally unvalidated criteria for group allocation in the Brazilian population. Future studies may be conducted to validate or develop new criteria for the commencement of end-of-life care.

## 5. Conclusions

Individuals with interstitial lung disease (ILD) with an indication for end-of-life care (EOLC) exhibit lower levels of physical activity in daily life, functional performance, peripheral muscle strength, quality of life, and worse sensations of dyspnea. There were no significant differences in respiratory muscle strength, body composition, sleep quality, daytime sleepiness, or symptoms of anxiety and depression. Understanding these findings contributes to the early management of debilitating functional symptoms and improves the quality of palliative care.

## Figures and Tables

**Table 1:** End-of-life care indications for individuals with interstitial lung diseases.

Indications of EOLC for Individuals with ILD
Two or more respiratory hospitalizations in the last year. Peripheral oxygen saturation < 88% at rest. Reduced exercise capacity (i.e., 6 min walk for a distance < 212 m). Diagnosis of pulmonary hypertension. Pulmonary function: FVC < 50% predicted, a decline in %FVC > 10% predicted, or a decline in D_L_CO > 15% in 6 months.

m = meters; FVC = Forced Vital Capacity; D_L_CO = Diffusing capacity for carbon monoxide.

**Table 2:** Sample characteristics.

Variables	ILD-EOLC (*n* = 14)	ILD-nEOLC (*n* = 44)	*p*
Sex, female (%)	8 (57%)	25 (59%)	0.89
Age, years	62 ± 10	60 ± 10	0.27
Time since diagnosis, months	60 [24–102]	72 [36–132]	0.18
Home oxygen therapy, *n*	6 (42%)	8 (18%)	0.02
Deaths, number	11 (78%)	3 (7%)	<0.0001
Time of death, months	3 [2–11]	7 [5–10]	0.14
Diagnosis			
Idiopathic pulmonary fibrosis, *n*	11 (79%)	17 (39%)	0.01
Connective-tissue-disease-associated ILD, *n*	3 (21%)	22 (50%)	0.07
Sarcoidosis, *n*	0 (0%)	2 (5%)	0.99
Asbestosis, *n*	0 (0%)	1 (2%)	0.99
Non-specific interstitial pneumonia, *n*	0 (0%)	1 (2%)	0.99
Other ILD, *n*	0 (0%)	1 (2%)	0.99
Disease Staging			
GAP I, %	1 (7%)	33 (75%)	
GAP II, %	8 (57%)	11 (25%)	
GAP III, %	5 (36%)	0 (0%)	<0.0001
Comorbidities, *n*			
0–3	4 (29%)	21 (48%)	
4–6	7 (50%)	11 (25%)	
≥7	3 (21%)	12 (27%)	0.20
Pharmacological treatment			
Antifibrotics	1 (7%)	1 (2%)	0.42
Corticosteroids	5 (35%)	17 (38%)	0.93
Immunosuppressants	5 (35%)	21 (47%)	0.54

% = percentage; ILD = interstitial lung disease; GAP = gender–age–physiology index.

**Table 3:** Criteria for end-of-life care indication.

Variables	ILD-EOLC (*n* = 14)	ILD-nEOLC (*n* = 44)	*p*
Hospitalized in the last year, *n*	11 (78%)	10 (22%)	0.0009
Peripheral oxygen saturation < 88% at rest, *n*	6 (42%)	5 (11%)	0.016
Diagnosis of pulmonary hypertension, *n*	1 (7%)	8 (18%)	0.43
Lung Function			
FVC, % of predicted	52 ± 24	77 ± 18	0.002
FEV_1_, % of predicted	56 ± 24	77 ± 18	0.003
D_L_CO, % of predicted	22 ± 10	50 ± 13	<0.0001
TLC, % of predicted	51 [34–70]	74 [64–93]	0.009
FRC, % of predicted	58 [43–95]	64 [56–81]	0.13
IC, % of predicted	51 [34–70]	77 [63–93]	0.002
RV, % of predicted	75 [54–88]	78 [54–111]	0.21
Exercise Capacity			
6MWT, meters	399 ± 106	468 ± 100	0.0004
6MWT, % of predicted	64 ± 20	87 ± 16	0.0005

% = percentage; ILD = interstitial lung disease; FVC = forced vital capacity; FEV_1_ = forced expiratory volume; D_L_CO = diffusing capacity of carbon monoxide; TLC = total lung capacity; FRC = functional residual capacity; IC = inspiratory capacity; RV = residual volume; 6MWT = six-minute walk test.

**Table 4:** Comparison of clinical outcomes.

Variables	ILD-EOLC (*n* = 14)	ILD-nEOLC (*n* = 44)	*p*
Physical Activity in Daily Life			
Steps, n/day	2727 ± 2243	5170 ± 2373	0.001
Light activity, min/day	194 ± 98	309 ± 103	0.001
Moderate-to-vigorous physical activity, min/day	1 [0.4–1]	10 [3–19]	0.0003
Time standing, hours/day	3.9 ± 1.2	5.3 ± 1.8	0.005
Time sitting, hours/day	6.8 ± 1.9	7.6 ± 1.7	0.14
Time lying down, hours/day	6.4 ± 1.6	4.4 ± 1.5	0.0004
Functional performance			
Timed-up-and-go usual, seconds	11.1 [9.4–13.2]	9.9 [9.4–11.5]	0.19
Timed-up-and-go usual,% of predicted	80 ± 15	86 ± 18	0.10
1 min sit-to-stand test, repetitions	20 ± 4	26 ± 7	0.01
1 min sit-to-stand test, % of predicted	35 ± 8	45 ± 13	0.005
4-metre gait speed, m/s	0.92 ± 0.21	1.05 ± 0.15	0.02
4-metre gait speed, % of predicted	90 ± 20	105 ± 13	0.007
Peripheral muscle strength			
Deltoid, MVIC (N)	91 [71–126]	118 [97–167]	0.01
Pectoralis major, MVIC (N)	78 [65–94]	112 [71–128]	0.02
Latissimus dorsi, MVIC (N)	52 [41–66]	79 [58–114]	0.008
Biceps brachii, MVIC (N)	177 [248–204]	201 [164–243]	0.09
Triceps brachii, MVIC (N)	107 [97–138]	138 [110–182]	0.008
Quadriceps femoris, MVIC (N)	237 [211–303]	319 [261–446]	0.005
Respiratory muscle strength			
Maximal inspiratory pressure, mmHg	71 ± 23	85 ± 35	0.14
Maximal inspiratory pressure, % of predicted	80 ± 30	92 ± 35	0.13
Maximal expiratory pressure, mmHg	99 ± 19	97 ± 44	0.28
Maximal expiratory pressure, % of predicted	110 ± 36	104 ± 40	0.42
Body composition			
BMI, kg/m^2^	25.2 ± 4.07	27.9 ± 5.5	0.08
Body fat percentage, %	25 ± 9	24 ± 9	0.35
Percentage of lean mass, %	42 ± 10	49 ± 10	0.08
Basal metabolic rate, kcal	1335 [−1162–1519]	1424 [1166–1677]	0.30
Health-related quality of life			
SGRQ-I totals, points	71 ± 15	50 ± 20	0.0009
SGRQ-I symptoms, points	72 ± 19	61 ± 35	0.08
SGRQ-I activity, points	84 ± 22	59 ± 28	0.001
SGRQ-I impacts, points	62 ± 15	44 ± 21	0.005
Dyspnea in daily life			
UCSD-SOBQ, points	63 [41–92]	37 [17–53]	0.006
MRC, points	4 [3–5]	2 [2–3]	0.001
Anxiety and depression symptoms			
HADS Anxiety, points	6 [4–8]	5 [3–8]	0.19
HADS Depression, points	5 [3–7]	4 [2–8]	0.49
Sleep quality and daytime sleepiness			
PSQI, points	7 [6–8]	8 [6–10]	0.20
ESS, points	5 [2–10]	6 [4–10]	0.15

Data expressed as mean ± standard deviation or median and interquartile range; % = percentage; m/s = meters per second; Min = minutes; MVIC = maximum voluntary isometric contraction; N = Newtons; mmHg = millimeters of mercury; Kcal = kilocalories; SGRQ-I = Saint George’s Respiratory Questionnaire for FPI individuals; UCSD-SOBQ = University of California San Diego Shortness of Breath Questionnaire; MRC = medical research council scale; PSQI = Pittsburgh Sleep Quality Index; ESS = Epworth Sleepiness Scale.

## Data Availability

The data presented in this study may be available upon reasonable request from the corresponding author.
